# Artificial Intelligence–Assisted ACR TI-RADS and Bethesda-like Risk Prediction for Indeterminate Thyroid Nodules: A Retrospective Proof-of-Concept Evaluation

**DOI:** 10.3390/diagnostics16213616

**Published:** 2026-11-02

**Authors:** Duzgun Yildirim, Deniz Esin Tekcan Sanli, Meltem Kursun, Burcu Bektas Gunes, Aytug Altundag, Leonardo Obinna Iheme, Mustafa Hurmuzli, Ahmet Necati Sanli, Eren Yılmaz, Filiz Celebi, Ayten Paşazade, Bülent Çolakoğlu, Enes Yerdes

**Affiliations:** 1 Department of Radiology, Acıbadem Taksim Hospital, Istanbul 34373, Turkey; drduzgunyildirim@gmail.com; 2 Department of Radiology, Faculty of Medicine, Gaziantep University, Gaziantep 27310, Turkey; tekcandenizesin@gmail.com; 3 Department of Radiology, Bucak State Hospital, Burdur 15300, Turkey; mmeltem.kursun@gmail.com; 4 Department of Computer Engineering, Gedik University, Istanbul 34876, Turkey; burcu.gunes@gedik.edu.tr; 5 Department of Otorhinolaryngology, School of Medicine, Biruni University, Istanbul 34295, Turkey; aaltundagkbb@gmail.com; 6 EPAM Systems Inc., Istanbul 34475, Turkey; leonardo.iheme@gmail.com; 7 Department of Radiology, Acibadem Kozyatagi Hospital, Istanbul 34734, Turkey; mhurmuzlu19@gmail.com (M.H.); pashazadeayten@gmail.com (A.P.); 8 Department of General Surgery, Abdulkadir Yuksel State Hospital, Gaziantep 27100, Turkey; 9 Department of Ear Nose and Throat Diseases Head and Neck Surgery, Medipol Acibadem Regional Hospital, Istanbul 34718, Turkey; yilmazeren09@gmail.com; 10 Department of Radiology, Faculty of Medicine, Yeditepe University, Istanbul 34718, Turkey; elbuken.filiz@gmail.com; 11 Department of Radiology, American Hospital, Istanbul 34365, Turkey; blntc@yahoo.com; 12 Department of Nuclear Medicine, Faculty of Medicine, Gaziantep University, Gaziantep 27310, Turkey; e.yerdes@gmail.com

**Keywords:** thyroid nodules, artificial intelligence, indeterminate cytology, ACR TI-RADS, Bethesda System for Reporting Thyroid Cytopathology, diagnostic accuracy, ultrasonography

## Abstract

**Background/Objectives**: Cytologically indeterminate thyroid nodules (Bethesda III–IV) remain a diagnostic challenge. We evaluated the TRAICK (version 1.9.4; TRAICK, Istanbul, Türkiye) artificial intelligence (AI) system, which generates an American College of Radiology Thyroid Imaging Reporting and Data System (ACR TI-RADS) category and an imaging-derived Bethesda-like risk category from gray-scale ultrasound images. **Methods**: This retrospective proof-of-concept study included 52 surgically resected nodules with Bethesda III (*n* = 29) or IV (*n* = 23) cytology, with histopathology of the index nodule as the reference standard. The two TRAICK outputs were analyzed separately, and the ACR TI-RADS output was compared with two radiologists using the McNemar and DeLong tests. **Results**: Twenty-three nodules (44.2%) were malignant on index-nodule histopathology. The TRAICK ACR TI-RADS output had an accuracy of 71.2% (95% CI 57.7–81.7) and an area under the ROC curve (AUC) of 0.762 (0.640–0.884). Its AUC did not differ significantly from that of Radiologist 1 (0.762 vs. 0.777, *p* = 0.783); specificity was higher (65.5% vs. 37.9%, *p* = 0.021), whereas sensitivity was lower without a statistically significant difference (78.3% vs. 91.3%, *p* = 0.250). Compared with Radiologist 2, the TRAICK ACR TI-RADS output had higher accuracy (71.2% vs. 51.9%, *p* = 0.013), specificity (65.5% vs. 24.1%, *p* < 0.001), and AUC (0.762 vs. 0.629, *p* = 0.042). The Bethesda-like output had an AUC of 0.822 (0.707–0.938), with a sensitivity of 60.9% and specificity of 86.2% at the V–VI threshold. In sensitivity analyses, the AUC of the ACR TI-RADS output did not differ significantly from that of Radiologist 1, whereas its AUC advantage over Radiologist 2 was not statistically significant when low-risk neoplasms were classified as malignant. **Conclusions**: In this small, selected surgical cohort, the TRAICK ACR TI-RADS output showed discrimination that did not differ significantly from that of the more accurate of two experienced radiologists, with higher specificity, and its Bethesda-like output showed promising discrimination. These findings support further study of TRAICK as a complementary aid for preoperative decision-making; independent prospective validation is needed.

## 1. Introduction

Thyroid nodules are highly prevalent in adults, and ultrasonography (US) is the primary modality for risk assessment. Risk stratification systems such as the American College of Radiology Thyroid Imaging Reporting and Data System (ACR TI-RADS), the European Thyroid Association Thyroid Imaging Reporting and Data System (EU-TIRADS), and the American Thyroid Association (ATA) guidelines were developed to standardize reporting and guide management [1,2,3]. However, they remain limited by interobserver variability and suboptimal sensitivity, particularly in nodules with indeterminate cytology [4,5].

Fine-needle aspiration biopsy (FNAB), interpreted according to the Bethesda System for Reporting Thyroid Cytopathology, remains the standard preoperative diagnostic method [6,7]. However, Bethesda categories III (atypia of undetermined significance, AUS) and IV (follicular neoplasm, FN) form a well-recognized diagnostic “gray zone”, with an expected risk of malignancy of roughly 13% to 34% [7]. As a result, many patients undergo repeat biopsies or diagnostic surgery, and a considerable proportion of resected nodules ultimately prove benign [6,7]. Such surgery is not without cost. Thyroidectomy carries risks of recurrent laryngeal nerve injury, hypoparathyroidism, and lifelong thyroid hormone replacement. Even among patients operated on for low-risk thyroid cancer, a notable proportion later report regret about their decision, particularly after postoperative complications [8]. Better preoperative risk information for indeterminate nodules could therefore help clinicians and patients weigh the benefits and harms of surgery more carefully.

Artificial intelligence (AI) has shown promising results in US-based thyroid nodule classification, with performance comparable to that of experienced radiologists in several studies and recent meta-analyses [9,10,11,12]. However, most AI approaches focus solely on imaging-based ACR TI-RADS classification, and few have been specifically evaluated in cytologically indeterminate nodules [13]. To explore this gap, we developed the TRAICK AI System (version 1.9.4; TRAICK, Istanbul, Türkiye). From gray-scale US images, it generates both an ACR TI-RADS category and an imaging-derived, Bethesda-like risk category. This second output is an imaging-based estimate of cytological risk, not a cytological diagnosis, and it is intended to complement FNAB rather than replace it.

In this retrospective proof-of-concept study, we aimed to evaluate the diagnostic performance of the TRAICK AI System in surgically resected, cytologically indeterminate thyroid nodules (Bethesda III–IV), using postoperative histopathology as the reference standard. We also compared its performance with that of two radiologists, and we described how its Bethesda-like output redistributed nodules within the indeterminate cytology categories.

## 2. Methods

### 2.1. Study Design and Reporting

**Figure 1:**
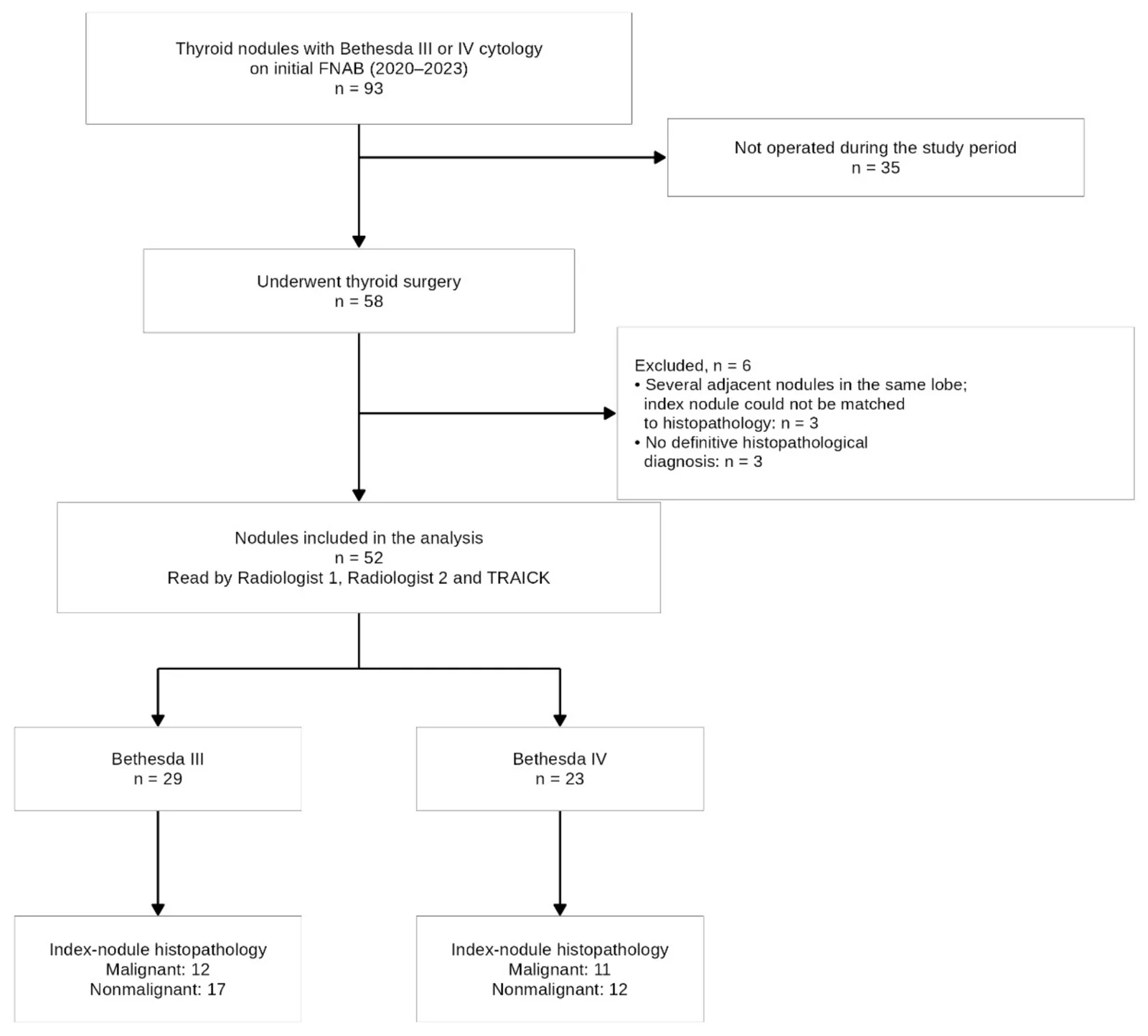
Flow of thyroid nodules through the study. FNAB, fine-needle aspiration biopsy.

This was a retrospective, single-center, proof-of-concept evaluation of an artificial intelligence (AI) system for ultrasound-based risk assessment of cytologically indeterminate thyroid nodules. It was not designed to assess clinical management or patient outcomes. The study was reported in line with the STARD-AI guideline for diagnostic accuracy studies using AI [14]; the completed checklist is provided as Supplementary File S1, and the flow of nodules through the study is shown in Figure 1. The study was approved by the Acıbadem University and Acıbadem Healthcare Institutions Medical Research Ethics Committee (Approval No: 2022-12/08). Because TRAICK was applied retrospectively to stored images, the index test could not cause adverse events. Complications of FNAB and surgery were not recorded for this study. The need for study-specific consent was waived because of the retrospective design; all patients had given written informed consent for FNAB as part of routine care.

### 2.2. Patient Selection

We reviewed all thyroid nodules with an initial fine-needle aspiration biopsy (FNAB) result of Bethesda category III (atypia of undetermined significance, AUS) or IV (follicular neoplasm, FN) at our institution between 2020 and 2023 (*n* = 93). Of these, 58 nodules subsequently underwent thyroid surgery, and 35 were not operated on during the study period. Surgery was performed for persistent indeterminate cytology on repeat FNAB, suspicious sonographic features, nodule size or growth, compressive symptoms, or patient preference. Nodules were eligible if they had a definitive postoperative histopathological diagnosis, preoperative gray-scale ultrasound images of adequate quality, and a histopathology report that could be matched to the sampled nodule. Six operated nodules were excluded: three because the lobe contained several adjacent nodules and the sampled nodule could not be matched to the histopathology report with confidence, and three because the histopathology report did not give a definitive benign or malignant diagnosis (the report described a lesion possibly corresponding to noninvasive follicular thyroid neoplasm with papillary-like nuclear features [NIFTP]). The final analysis therefore included 52 nodules (Bethesda III, *n* = 29; Bethesda IV, *n* = 23) from 52 patients (one nodule per patient).

Nodules with Bethesda I, II, V, or VI cytology were not included, because the aim was to study the indeterminate categories, in which preoperative decision-making is most difficult. Because only operated nodules could be verified by histopathology, the study population is a selected surgical cohort. It is likely to contain a higher proportion of malignant nodules than the full population of patients with Bethesda III–IV cytology, and the nonmalignant nodules that reached surgery were probably selected on clinical or sonographic grounds. This spectrum may affect sensitivity and specificity, not only the predictive values, and it was taken into account when interpreting the results.

Age, sex, nodule size (mm), cytological Bethesda category, and final histopathological diagnosis were retrieved from institutional records.

### 2.3. Ultrasound Examination and FNAB

Ultrasound examinations were performed with a GE Logiq S8 system (GE HealthCare, Chicago, IL, USA) and a 9L linear probe using a standardized institutional protocol. At the time of clinical care, all nodules were examined by one radiologist (D.Y., 25 years of experience in thyroid imaging) and categorized according to the American College of Radiology Thyroid Imaging Reporting and Data System (ACR TI-RADS) [1].

Nodules meeting ACR TI-RADS criteria for biopsy underwent ultrasound-guided FNAB. Several aspirates were obtained from each nodule, and sample adequacy was confirmed on site by a cytopathologist. Cytological evaluation was performed using conventional smears and ThinPrep (Hologic, Marlborough, MA, USA) cell blocks. Results were reported using the Bethesda System for Reporting Thyroid Cytopathology; category names in this report follow the 2023 edition [7]. The cytopathologist had no access to TRAICK outputs.

### 2.4. Image Selection

During routine examinations, an average of four to five gray-scale images were stored for each nodule. For this study, one image per nodule was retrieved from the picture archiving and communication system (PACS). Only pure gray-scale images were eligible, that is, images without caliper measurements, Doppler or elastography overlays, color coding, on-screen markers, or visible needle artifacts (File S2). Images were selected by D.Y., who was blinded to the cytological and histopathological results. The same single image was used for both radiologist readings and for the TRAICK analysis.

### 2.5. Radiologist Reading

Two radiologists (D.Y., 25 years of experience; D.E.T.S., 15 years of experience in thyroid imaging) independently re-read the selected static image of each nodule and assigned an ACR TI-RADS category to each nodule. They did not have access to the full ultrasound cine loops or the original clinical reports. Both readers were blinded to cytology, histopathology, and TRAICK outputs, and cases were presented in random order. Because D.Y. had performed the original clinical examinations, a washout interval of at least 3 months separated the clinical examination from the study reading.

D.Y. was also involved in the development of the TRAICK system (see Conflicts of Interest). He did not run the TRAICK analysis or the statistical analysis. Radiologist 2 (D.E.T.S.) has no affiliation with TRAICK, and all comparisons are reported separately for each reader so that the comparison with the independent reader can be assessed on its own.

### 2.6. Histopathological Reference Standard

The histopathology of the index nodule itself served as the reference standard for all analyses. Surgical pathology reports were reviewed by an experienced endocrine pathologist who was blinded to the radiologist readings and TRAICK outputs. Diagnoses followed the World Health Organization (WHO) Classification of Tumours, 5th edition [15]. A nodule was classified as malignant when carcinoma was present in the index nodule: papillary thyroid carcinoma (including the invasive encapsulated follicular variant), follicular thyroid carcinoma, or oncocytic carcinoma (including minimally invasive tumors). All other diagnoses were classified as nonmalignant, including benign lesions (follicular nodular disease, follicular or oncocytic adenoma, and lymphocytic thyroiditis) and the low-risk neoplasms of the 2022 WHO classification.

Seven of the 52 included nodules were low-risk neoplasms: six NIFTP and one well-differentiated tumor of uncertain malignant potential (WT-UMP). The WHO Classification of Tumours (5th edition) does not consider these tumors carcinomas; they are low-risk neoplasms that are usually treated by lobectomy alone [15]. In the primary analysis, all seven were therefore classified as nonmalignant. Because the handling of these lesions can change the results, the analysis was repeated with all of them classified as malignant (sensitivity analysis).

### 2.7. Imaging–Pathology Correlation in Multifocal Disease

In patients with more than one nodule, the index nodule was the one that had undergone FNAB with a Bethesda III–IV result. Its correspondence with the histopathology report was established using lobe, location, and size. Glands with several adjacent nodules in the same lobe, in which this match could not be made with confidence, were excluded (*n* = 3). Four of the 52 included patients had more than one nodule on ultrasound; in each of them, the index nodule could be matched to the histopathology report. Among the 52 included nodules, the histopathology report described papillary microcarcinoma outside the index nodule in six cases, in the contralateral lobe or as separate foci elsewhere in the gland. On pathology review, the index nodule itself was benign in five of these cases and a WT-UMP in one. Because the reference standard was the histology of the index nodule, these six nodules were classified as nonmalignant in the primary analysis. To assess the effect of this decision, the analysis was repeated after excluding these six nodules, so that only nodules with a secure match between imaging, cytology, and histology were analyzed (*n* = 46), and with the original database coding, in which they were counted as malignant.

### 2.8. TRAICK AI System

The TRAICK AI System (version 1.9.4) generates two separate outputs from gray-scale ultrasound images: (1) an ACR TI-RADS category and (2) an imaging-derived, Bethesda-like risk category (II–VI). The Bethesda-like output is an estimate of cytological risk based on image features. It is not a cytological diagnosis, and it is intended to complement FNAB rather than replace it. The system reports final categories only; it does not export a continuous malignancy probability. It is intended for use by radiologists and other clinicians who perform or interpret thyroid ultrasound, as an aid to their own ACR TI-RADS assessment, and does not require technical expertise to operate.

The system was developed using 9185 gray-scale ultrasound images from several institutions, including hospitals of the healthcare group to which our institution belongs (File S2). The development images were split into training, validation, and test sets (approximately 52%, 22%, and 26%). The test set was separated at the patient level, so that no test patient contributed images to training or validation; the validation set was drawn at random from the remaining images. This applies to the ACR TI-RADS feature classifiers; the segmentation model was developed with a separate, image-level split (File S2). None of the 52 nodules or patients in the present cohort contributed images to the development dataset, and the cohort was not used for training, validation, or testing of the model. Because hospitals of the same group, including our own, contributed development images from other patients, the present analysis is an evaluation in patients independent of the development data, rather than a fully external evaluation. A YOLOv8-based module first locates and segments the nodule. Five separate classifiers then predict the five ACR TI-RADS features (composition, echogenicity, shape, margin, and echogenic foci). The feature-level outputs are converted into ACR TI-RADS points and summed to give the final ACR TI-RADS category using the standard ACR thresholds. The Bethesda-like module was trained separately using cytological and histopathological labels; for Bethesda IV nodules in the development data, labels were harmonized with the postoperative diagnosis. Full details of the development dataset, architecture, training, and feature-level performance are given in File S2.

**Figure 2:**
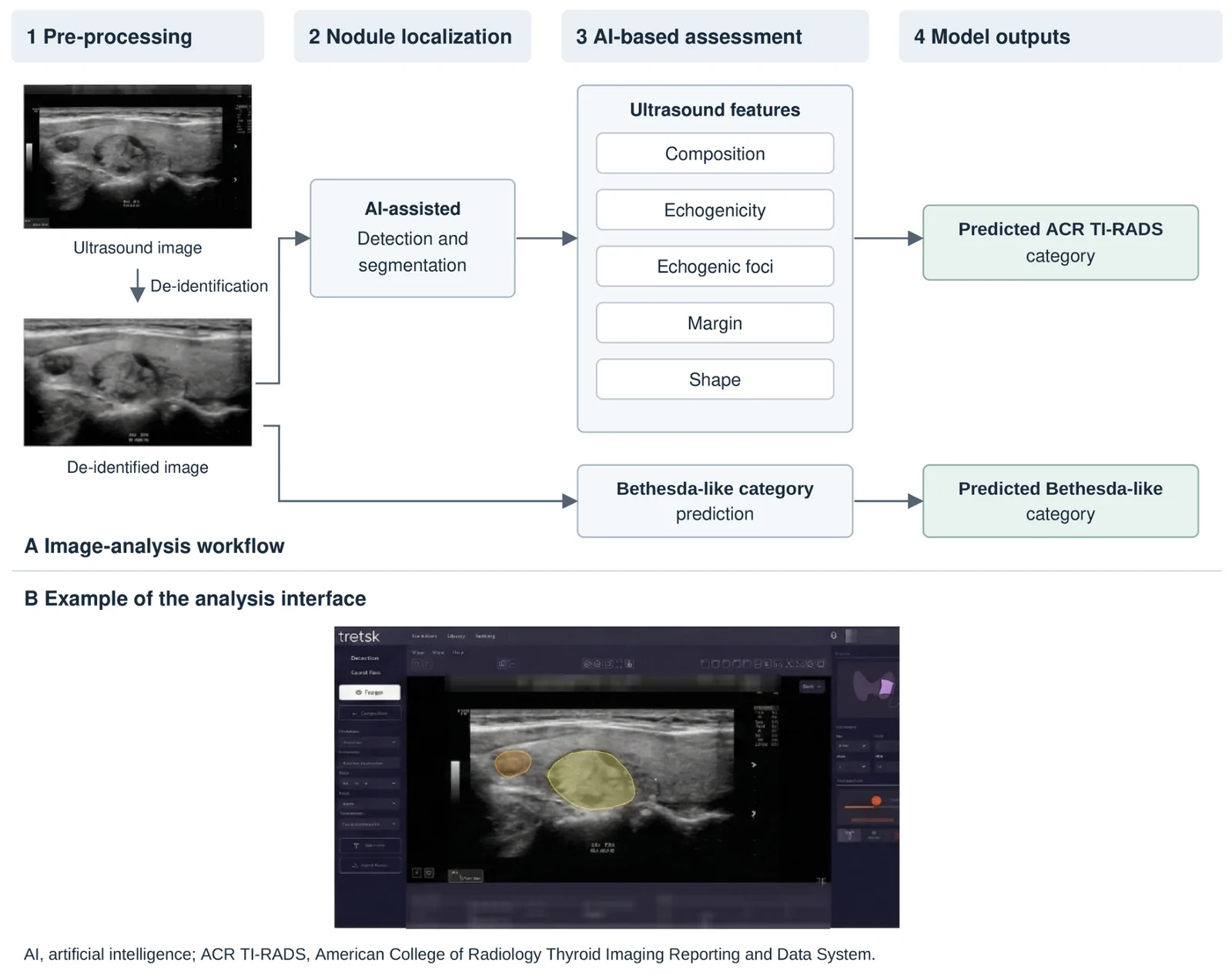
Overview of the TRAICK AI System. (**A**) Image-analysis workflow: pre-processing and de-identification, AI-assisted nodule detection and segmentation, assessment of the five ACR TI-RADS features, and the two model outputs (ACR TI-RADS category and Bethesda-like category). (**B**) Example of the analysis interface.

**Figure 3:**
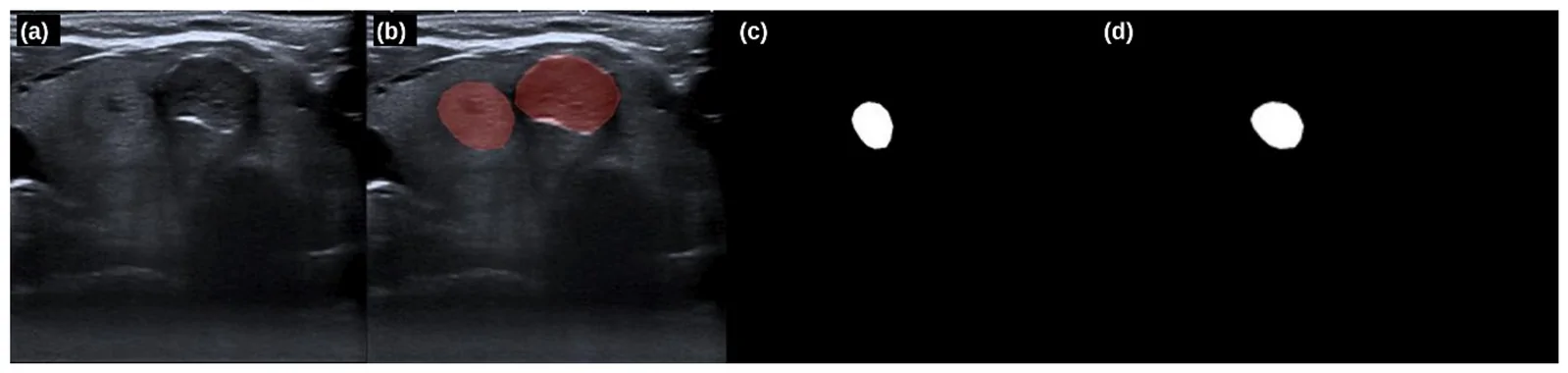
Extraction of nodule masks: (**a**) original ultrasound image; (**b**) annotated image; (**c**) mask of the first nodule; (**d**) mask of the second nodule.

**Figure 4:**
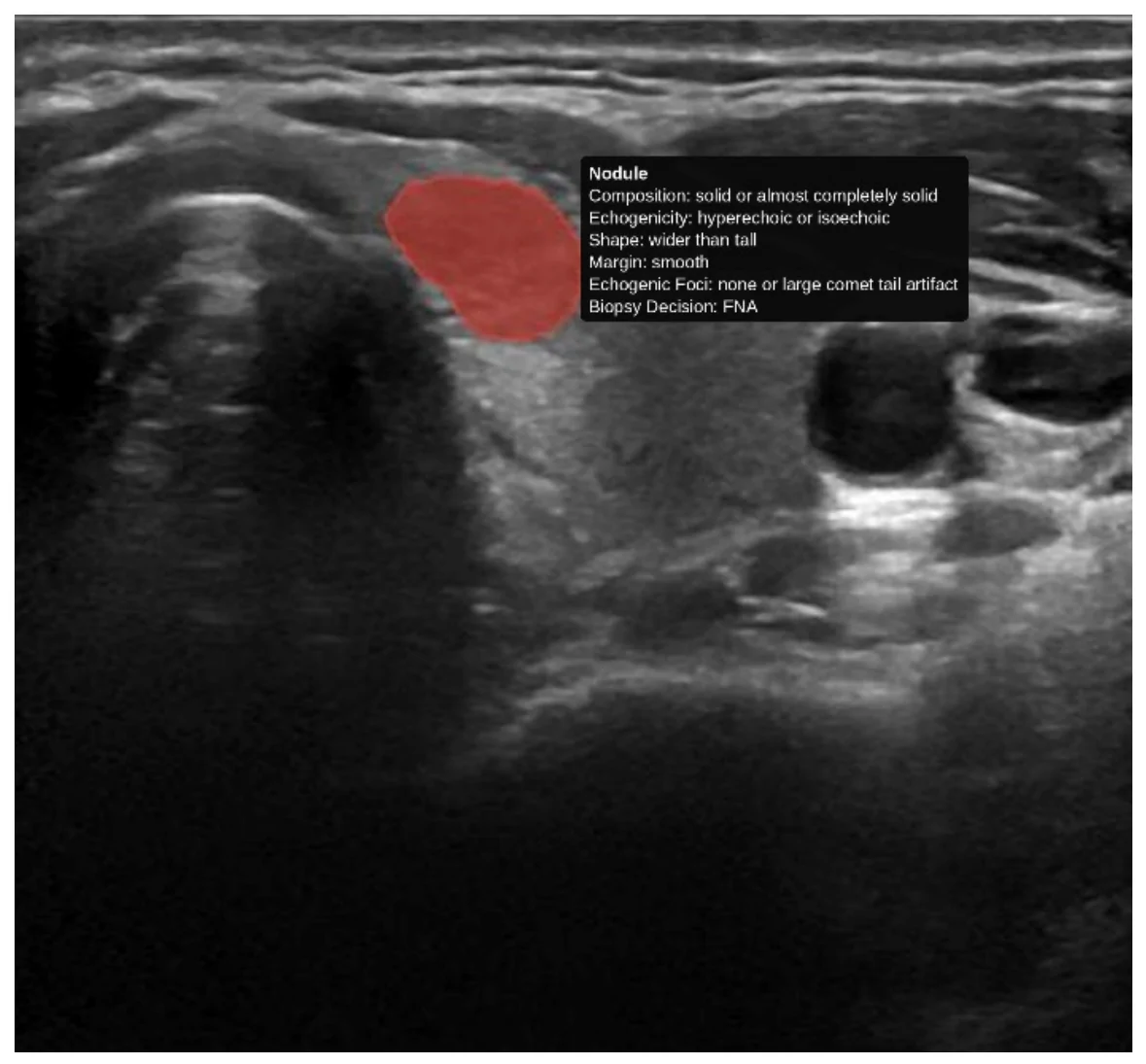
Representative TRAICK AI System output for a thyroid nodule, showing automated segmentation and feature-level ACR TI-RADS classification with a biopsy recommendation. The text annotations of the original output were re-typeset for legibility.

The model was not retrained, fine-tuned, or recalibrated on these data, and no thresholds were adjusted. The selected images were processed through the standard inference pipeline (normalization, resizing, and removal of on-screen annotations), and the system’s final categorical outputs were recorded. The overall architecture and workflow of the system are shown in Figure 2, an example of automated nodule segmentation in Figure 3, and a representative output in Figure 4.

### 2.9. Definitions and Analysis Plan

The two TRAICK outputs were analyzed separately, as they address different classification tasks:

ACR TI-RADS output (TRAICK and both radiologists): TR4–TR5 was considered test-positive for malignancy, and TR1–TR3 test-negative.

Bethesda-like output (TRAICK only): categories V–VI were considered test-positive, and II–IV test-negative.

Both thresholds were prespecified and correspond to commonly used clinical cut-offs. They were not optimized on the study data.

Because all nodules had indeterminate (Bethesda III–IV) cytology by design, FNAB cytology was not treated as a binary diagnostic comparator. Instead, we described how the TRAICK Bethesda-like output redistributed nodules within each cytological category (reclassification analysis). Results were stratified by final histopathology, so that it could be seen whether upward or downward reclassification agreed with the final diagnosis.

### 2.10. Statistical Analysis

Diagnostic performance was assessed at the nodule level; because one nodule was included per patient, nodule-level and patient-level analyses were identical. For each reader and output, sensitivity, specificity, accuracy, positive predictive value (PPV), and negative predictive value (NPV) were calculated against histopathology and reported with numerators, denominators, and 95% confidence intervals (CIs) using the Wilson score method. Agreement with histopathology and agreement between readers were assessed with Cohen’s kappa (κ). The 95% CIs for κ were based on the large-sample standard error of Fleiss, Cohen, and Everitt, and κ values were interpreted on the Landis and Koch scale. For agreement between readers, the main estimate was unweighted κ across ACR TI-RADS categories; linearly weighted κ and κ for the binary (≥TR4) classification are also reported. All 52 nodules had results for both radiologists, both TRAICK outputs, and the reference standard; there were no missing or indeterminate test results.

Receiver operating characteristic (ROC) curves were constructed directly from the ordinal categories (ACR TI-RADS categories for the radiologists and TRAICK; Bethesda-like categories II–VI for the TRAICK Bethesda-like output), so that each point on a curve corresponds to one category cut-off. Areas under the curve (AUCs) were reported with 95% CIs using the DeLong method.

Paired comparisons on the same nodules used the exact McNemar test for accuracy (all nodules), sensitivity (malignant nodules only), and specificity (nonmalignant nodules only). AUCs were compared using the DeLong test for paired ROC curves. Malignancy rates across TRAICK ACR TI-RADS categories within each cytological Bethesda category were reported with Wilson 95% CIs, and a trend across categories was tested with the Cochran–Armitage test. Nodule size was compared between malignant and nonmalignant nodules with the Mann–Whitney U test.

Three sensitivity analyses were performed: (i) classifying the low-risk neoplasms (six NIFTP and one WT-UMP) as malignant; (ii) excluding the six nodules with carcinoma outside the index nodule (secure imaging–pathology match, *n* = 46); and (iii) using the original database coding, in which carcinoma found anywhere in the gland was counted as malignant and NIFTP cases were coded as recorded at data collection (34 malignant, 18 benign). These analyses were not prespecified in a study protocol and should be regarded as exploratory.

No formal sample size calculation was performed, as this was a proof-of-concept study based on all eligible cases in the study period. The paired comparisons were therefore considered exploratory, and no adjustment for multiple testing was made. All tests were two-sided, and *p* < 0.05 was considered statistically significant. Analyses were performed in R version 4.3.3 (R Foundation for Statistical Computing, Vienna, Austria) using the pROC (version 1.18.5), readxl (1.4.3), ggplot2 (3.4.4), and patchwork (1.2.0) packages; Wilson intervals, κ standard errors, and exact McNemar tests were coded in base R.

## 3. Results

### 3.1. Study Population

Of 93 nodules with Bethesda III–IV cytology during the study period, 58 underwent surgery and 52 met the inclusion criteria (Figure 1). Twenty-nine nodules (55.8%) had Bethesda III and 23 (44.2%) had Bethesda IV cytology. Each of the 52 nodules came from a different patient. The patients were 40 women (76.9%) and 12 men, with a mean age of 48.0 ± 8.9 years (range 32–65). Mean nodule size was 16.8 ± 9.8 mm (median 15 mm, interquartile range 10–21). The mean interval between the ultrasound examination and surgery was approximately 2 months.

On histopathology of the index nodule, 23 nodules (44.2%) were classified as malignant and 29 (55.8%) as nonmalignant. The malignancy rate was 41.4% (12/29) among Bethesda III nodules and 47.8% (11/23) among Bethesda IV nodules. The malignant group included papillary thyroid carcinoma (*n* = 15), the invasive encapsulated follicular variant of papillary thyroid carcinoma (*n* = 6), minimally invasive follicular carcinoma (*n* = 1), and minimally invasive oncocytic carcinoma (*n* = 1). The nonmalignant group included benign lesions (follicular nodular disease, follicular or oncocytic adenoma, or lymphocytic thyroiditis; *n* = 22), NIFTP (*n* = 6), and WT-UMP (*n* = 1). In six nonmalignant nodules, papillary microcarcinoma was present elsewhere in the gland. Malignant nodules were smaller than nonmalignant nodules (median 11 vs. 20 mm; *p* = 0.002).

### 3.2. Distribution of Categories

**Figure 5:**
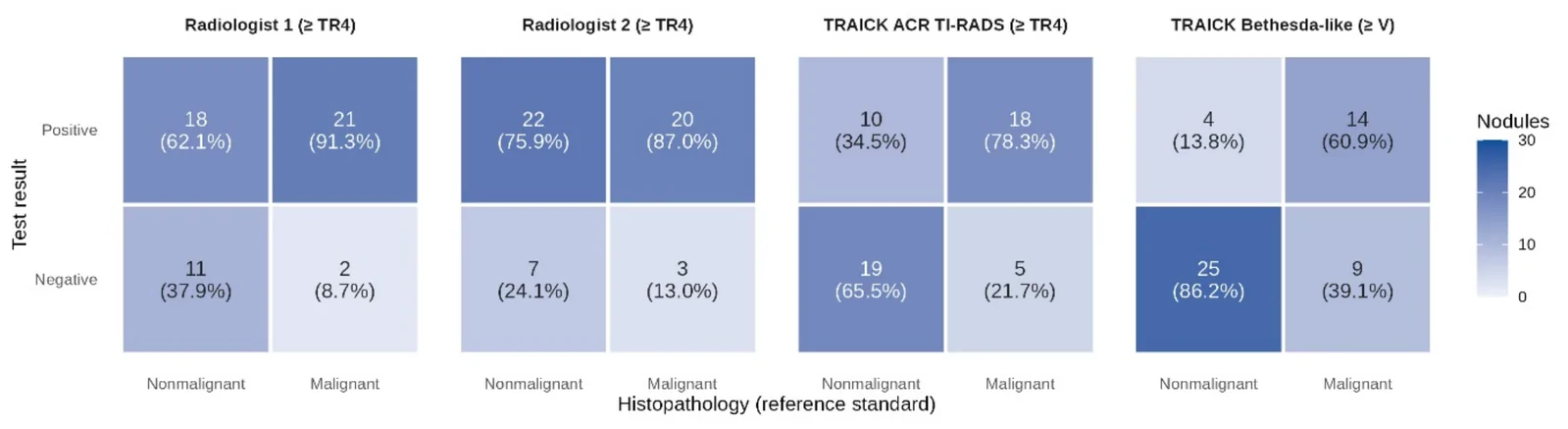
Confusion matrices for Radiologist 1, Radiologist 2, and the TRAICK ACR TI-RADS output (test-positive: TR4–TR5), and for the TRAICK Bethesda-like output (test-positive: categories V–VI), with index-nodule histopathology as the reference standard (*n* = 52; 23 malignant, 29 nonmalignant). Each cell shows the number of nodules and, in parentheses, the percentage of the histopathology column. All panels use the same color scale.

Radiologist 1 assigned TR3 to 13 nodules, TR4 to 25, and TR5 to 14; Radiologist 2 assigned TR3 to 10, TR4 to 28, and TR5 to 14. The TRAICK ACR TI-RADS output was TR2 in 1 nodule, TR3 in 23, TR4 in 20, and TR5 in 8. The TRAICK Bethesda-like output was category II in 13 nodules, III in 7, IV in 14, V in 9, and VI in 9. The resulting confusion matrices against histopathology are shown in Figure 5.

### 3.3. Diagnostic Performance of ACR TI-RADS Assessments

Diagnostic performance is summarized in Table 1. At the ≥TR4 threshold, the TRAICK ACR TI-RADS output classified 37 of 52 nodules correctly (accuracy 71.2%, 95% CI 57.7–81.7), with a sensitivity of 78.3% (18/23; 58.1–90.3) and a specificity of 65.5% (19/29; 47.3–80.1). Radiologist 1 had an accuracy of 61.5% (32/52; 48.0–73.5), a sensitivity of 91.3% (21/23; 73.2–97.6), and a specificity of 37.9% (11/29; 22.7–56.0). Radiologist 2 had an accuracy of 51.9% (27/52; 38.7–64.9), a sensitivity of 87.0% (20/23; 67.9–95.5), and a specificity of 24.1% (7/29; 12.2–42.1). Agreement with histopathology was moderate for TRAICK (κ = 0.428, 95% CI 0.188–0.668), fair for Radiologist 1 (κ = 0.273, 0.067–0.479), and slight for Radiologist 2 (κ = 0.102, −0.091 to 0.296).

**Figure 6:**
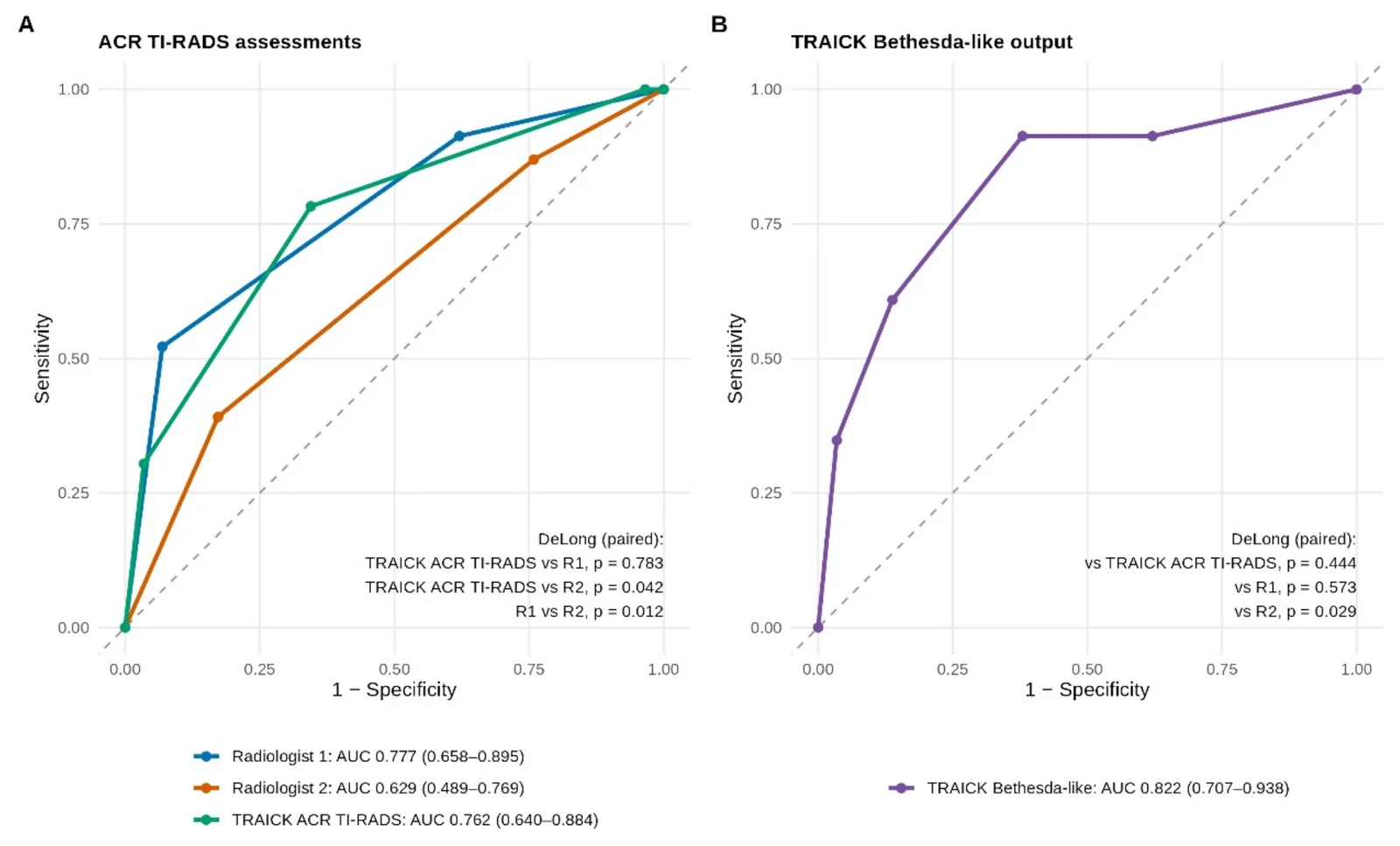
Receiver operating characteristic curves. (**A**) ACR TI-RADS assessments by Radiologist 1, Radiologist 2, and TRAICK. (**B**) TRAICK Bethesda-like output. Curves were drawn from the ordinal categories; each point corresponds to one category cut-off. AUCs are given with 95% confidence intervals (DeLong method), and *p* values are from the paired DeLong test. The dashed diagonal line indicates chance-level discrimination (AUC = 0.5).

The AUC was 0.762 (95% CI 0.640–0.884) for the TRAICK ACR TI-RADS output, 0.777 (0.658–0.895) for Radiologist 1, and 0.629 (0.489–0.769) for Radiologist 2 (Figure 6A).

### 3.4. Paired Comparisons

All paired comparisons are given in Supplementary Table S1. The TRAICK ACR TI-RADS output and Radiologist 1 did not differ significantly in accuracy (71.2% vs. 61.5%; McNemar *p* = 0.267) or AUC (0.762 vs. 0.777; DeLong *p* = 0.783). TRAICK had a higher specificity than Radiologist 1 (65.5% vs. 37.9%; *p* = 0.021), and its lower sensitivity (78.3% vs. 91.3%; *p* = 0.250) was not statistically significant. Radiologist 1 identified three malignant nodules that TRAICK classified as TR3, whereas TRAICK identified no malignant nodule that Radiologist 1 missed.

Compared with Radiologist 2, the TRAICK ACR TI-RADS output had a higher accuracy (71.2% vs. 51.9%; *p* = 0.013), a higher specificity (65.5% vs. 24.1%; *p* < 0.001), and a higher AUC (0.762 vs. 0.629; *p* = 0.042), with no significant difference in sensitivity (78.3% vs. 87.0%; *p* = 0.500). Radiologist 1 had a higher AUC than Radiologist 2 (*p* = 0.012), but their accuracy did not differ significantly (*p* = 0.125).

### 3.5. Diagnostic Performance of the TRAICK Bethesda-like Output

At the prespecified threshold (categories V–VI), the Bethesda-like output was positive in 18 nodules, 14 of which were malignant. Sensitivity was 60.9% (14/23; 95% CI 40.8–77.8), specificity 86.2% (25/29; 69.4–94.5), PPV 77.8% (14/18; 54.8–91.0), NPV 73.5% (25/34; 56.9–85.4), and accuracy 75.0% (39/52; 61.8–84.8), with moderate agreement with histopathology (κ = 0.482, 0.244–0.719) (Table 1, Figure 5).

Across all categories, the AUC of the Bethesda-like output was 0.822 (95% CI 0.707–0.938) (Figure 6B). It was higher than the AUC of Radiologist 2 (*p* = 0.029), whereas the differences from Radiologist 1 (*p* = 0.573) and from the TRAICK ACR TI-RADS output (*p* = 0.444) were not statistically significant. The malignancy rate was 15.4% (2/13; 95% CI 4.3–42.2) in category II, 0% (0/7; 0–35.4) in category III, 50.0% (7/14; 26.8–73.2) in category IV, 66.7% (6/9; 35.4–87.9) in category V, and 88.9% (8/9; 56.5–98.0) in category VI. The seven malignant nodules placed in category IV were counted as test-negative at the V–VI threshold.

### 3.6. Agreement Between Readers

Agreement in ACR TI-RADS categories (Table 2) was moderate between the two radiologists (κ = 0.411, 95% CI 0.194–0.628) and fair between TRAICK and Radiologist 1 (κ = 0.391, 0.196–0.586) and between TRAICK and Radiologist 2 (κ = 0.336, 0.150–0.523). Linearly weighted κ values were 0.506, 0.496, and 0.379, respectively.

### 3.7. Reclassification Within Indeterminate Cytology

**Figure 7:**
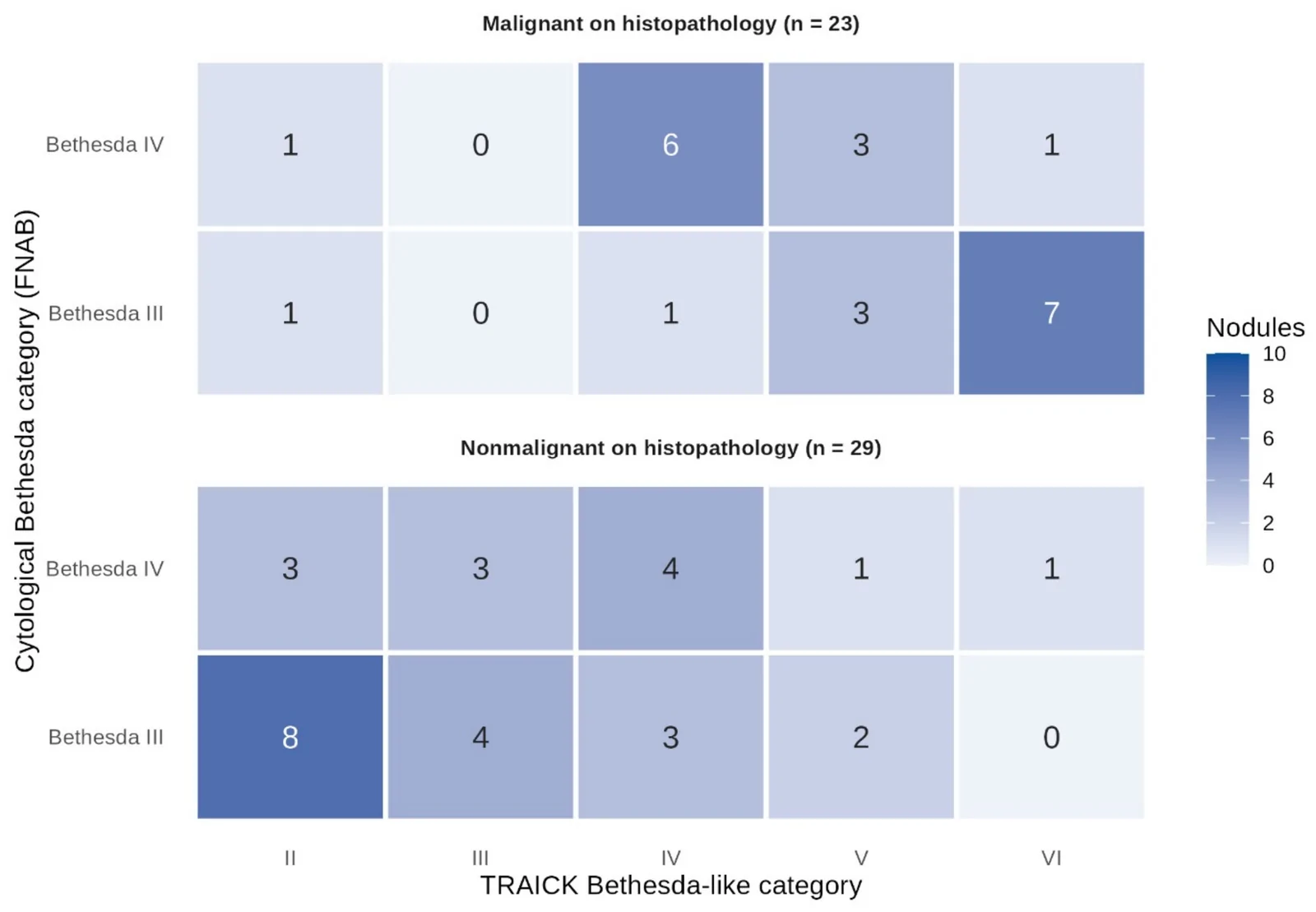
Reclassification heat map of cytologically indeterminate nodules by the TRAICK Bethesda-like output. Rows show the cytological Bethesda category (FNAB) and columns the TRAICK Bethesda-like category, separately for nodules that were malignant (*n* = 23) and nonmalignant (*n* = 29) on index-nodule histopathology. Each cell shows the number of nodules, and darker shading indicates a higher count.

The distribution of TRAICK Bethesda-like categories within each cytological category, by index-nodule histopathology, is shown in Table 3 and Figure 7. Of the 29 Bethesda III nodules (12 malignant, 17 nonmalignant), TRAICK placed 12 in categories V–VI (10 malignant, 2 nonmalignant), 4 in category IV (1 malignant, 3 nonmalignant), and 13 in categories II–III (1 malignant, 12 nonmalignant). Of the 23 Bethesda IV nodules (11 malignant, 12 nonmalignant), TRAICK placed 6 in categories V–VI (4 malignant, 2 nonmalignant), 10 in category IV (6 malignant, 4 nonmalignant), and 7 in categories II–III (1 malignant, 6 nonmalignant).

Overall, of the 23 malignant nodules, 14 (60.9%) were placed in categories V–VI, 7 (30.4%) in category IV, 2 (8.7%) in category II, and none in category III. Of the 29 nonmalignant nodules, 11 (37.9%) were placed in category II, 7 (24.1%) in category III, 7 (24.1%) in category IV, and 4 (13.8%) in categories V–VI. These findings describe how the imaging-derived categories redistributed nodules within an indeterminate cytology cohort; they are not a comparison of diagnostic accuracy between TRAICK and FNAB.

### 3.8. Malignancy Rates by TRAICK ACR TI-RADS Category Within Cytological Categories

Among Bethesda III nodules, the malignancy rate was 8.3% (1/12; 95% CI 1.5–35.4) for TR3, 50.0% (6/12; 25.4–74.6) for TR4, and 100% (5/5; 56.6–100) for TR5 (*p* for trend < 0.001). Among Bethesda IV nodules, the single TR2 nodule was nonmalignant, and the malignancy rate was 36.4% (4/11; 15.2–64.6) for TR3, 62.5% (5/8; 30.6–86.3) for TR4, and 66.7% (2/3; 20.8–93.9) for TR5 (*p* for trend = 0.132) (Table 4). Seven of the eight nodules assigned TR5 by TRAICK were malignant (87.5%; 95% CI 52.9–97.8). Several subgroups contained fewer than 10 nodules, and their CIs were wide.

### 3.9. Sensitivity Analyses

The sensitivity analyses are presented in Supplementary Table S2. When the low-risk neoplasms (six NIFTP and one WT-UMP) were classified as malignant (30 malignant, 22 nonmalignant), the AUC decreased for both radiologists and the TRAICK ACR TI-RADS output: 0.654 (95% CI 0.517–0.791) for the TRAICK ACR TI-RADS output, 0.688 (0.556–0.820) for Radiologist 1 (*p* = 0.592), and 0.526 (0.382–0.669) for Radiologist 2 (*p* = 0.081); the AUC of the Bethesda-like output was 0.838 (0.731–0.945).

After excluding the six nodules with carcinoma outside the index nodule (*n* = 46; 23 malignant, 23 nonmalignant), the AUCs were 0.820 (0.710–0.931) for the TRAICK ACR TI-RADS output, 0.802 (0.685–0.918) for Radiologist 1 (*p* = 0.732), and 0.634 (0.487–0.782) for Radiologist 2 (*p* = 0.006), and 0.851 (0.734–0.968) for the Bethesda-like output. With the original database coding (34 malignant, 18 benign), the corresponding AUCs were 0.776 (0.663–0.889), 0.731 (0.605–0.858; *p* = 0.494), 0.549 (0.405–0.693; *p* = 0.001), and 0.907 (0.816–0.998). In all three analyses, the TRAICK ACR TI-RADS output did not differ significantly from Radiologist 1, its AUC was higher than that of Radiologist 2 in two of the three analyses, and the Bethesda-like output had the highest AUC (range 0.838–0.907).

## 4. Discussion

### 4.1. Principal Findings

In this retrospective proof-of-concept study of 52 surgically resected thyroid nodules with Bethesda III–IV cytology, with the histopathology of the index nodule as the reference standard, the AUC of the TRAICK ACR TI-RADS output did not differ significantly from that of the more accurate of two radiologists (0.762 vs. 0.777), and TRAICK had a higher accuracy, specificity, and AUC than the second radiologist. Compared with Radiologist 1, TRAICK had a higher specificity (65.5% vs. 37.9%), while its lower sensitivity (78.3% vs. 91.3%) and the difference in accuracy were not statistically significant. The Bethesda-like output had an AUC of 0.822, and at the prespecified V–VI threshold it identified 14 of the 23 malignant nodules. These findings were broadly consistent in sensitivity analyses addressing the classification of low-risk neoplasms and of carcinoma outside the index nodule, although AUCs decreased for the radiologists and the TRAICK ACR TI-RADS output when low-risk neoplasms were classified as malignant, and the AUC advantage of TRAICK over Radiologist 2 was then not statistically significant, whereas the AUC of the Bethesda-like output increased slightly from 0.822 to 0.838. Given the small and selected cohort, they should be regarded as hypothesis-generating.

### 4.2. ACR TI-RADS Output Compared with the Radiologists

The main difference between TRAICK and the radiologists was in specificity. Both radiologists classified most nodules as TR4 or TR5, which resulted in high sensitivity but low specificity (37.9% and 24.1%). This should be interpreted with caution. In a surgical cohort of indeterminate nodules, the nonmalignant nodules that reach surgery are likely to have been selected because they appeared suspicious clinically or on ultrasound. Such nodules are harder to recognize as nonmalignant, and the low specificity of the radiologists may partly reflect this selection rather than a general tendency to overcall. The large difference between our two readers is consistent with the substantial interobserver variability reported for ultrasound assessment of thyroid nodules [16], including ACR TI-RADS assessment of cytologically indeterminate nodules [4].

TRAICK assigned fewer nonmalignant nodules to suspicious categories than either radiologist, but it did not detect more cancers. Radiologist 1 identified three malignant nodules that TRAICK classified as TR3, whereas TRAICK identified no malignant nodule that Radiologist 1 missed. Conversely, TRAICK classified as TR2–TR3 nine nonmalignant nodules that Radiologist 1 assigned to TR4–TR5, while the reverse occurred in one nodule. The potential contribution of TRAICK in this setting therefore lies in specificity rather than sensitivity. Whether this would translate into fewer unnecessary operations cannot be judged from the present design.

Two aspects of the reader comparison limit its interpretation. First, only two radiologists took part, and Radiologist 1 (D.Y.) was involved in the development of TRAICK and had performed the original clinical examinations. Although a washout interval was used and the reader was blinded to TRAICK outputs, full independence cannot be ensured. TRAICK’s AUC was significantly higher than that of the independent reader (Radiologist 2), but this reader’s agreement with histopathology was low (κ = 0.102), so this comparison mainly illustrates variability between readers. Second, both radiologists assessed the same single static image per nodule as TRAICK, without cine loops or clinical information. This ensured that the readers and the AI system had the same information, but it differs from routine practice, in which radiologists examine the nodule in real time. Our results therefore describe a comparison with two readers in this dataset and should not be generalized to radiologists as a group. Future validation studies should include multiple readers who are fully independent of the development team.

Agreement between TRAICK and each radiologist (κ = 0.391 and 0.336) was in the same range as agreement between the two radiologists (κ = 0.411). TRAICK did not simply reproduce either reader’s classification; however, this finding alone does not show that it is more reproducible than human readers, which would require repeated assessment of the same nodules on different images or ultrasound systems.

### 4.3. Bethesda-like Output and Reclassification Within Indeterminate Cytology

The Bethesda-like output is an imaging-derived risk category, not a cytological diagnosis. Its malignancy rates rose from category III to category VI (from 0% to 88.9%), but category II did not follow this order (2 of 13 nodules malignant), and category IV contained equal numbers of malignant and nonmalignant nodules. Because category IV was counted as test-negative at the prespecified V–VI threshold, the seven malignant nodules in this category were missed, and sensitivity at this threshold was 60.9%. The categories of this output therefore do not correspond directly to cytological Bethesda categories in terms of malignancy risk, and their clinical use, including the choice of threshold, needs to be defined in independent data rather than in this cohort. In the development data, labels for Bethesda IV nodules were harmonized with the postoperative diagnosis (File S2; this may have influenced how this output separates category IV from the higher categories and is a further reason for additional external validation of this output.

Within the cytological Bethesda III group, TRAICK placed 10 of 12 malignant nodules and only 2 of 17 nonmalignant nodules in categories V–VI, and 12 of 17 nonmalignant nodules in categories II–III. This suggests that imaging-based information may help to separate higher- from lower-risk nodules within the same indeterminate cytological category. However, these findings describe reclassification within an indeterminate cohort and should not be read as evidence that TRAICK outperforms FNAB. Bethesda III and IV are indeterminate rather than negative results, and nodules with Bethesda II, V, or VI cytology were not included in this study.

### 4.4. Malignancy Rates Across TRAICK ACR TI-RADS Categories

Within both cytological groups, the malignancy rate increased with the TRAICK ACR TI-RADS category, and seven of the eight nodules assigned TR5 were malignant. This pattern is consistent with the diagnostic value of ACR TI-RADS features in indeterminate nodules. However, the subgroups were small and the confidence intervals wide: the lower limit of the 95% CI for the TR5 malignancy rate was 52.9%, and the trend was not statistically significant among Bethesda IV nodules. These rates indicate diagnostic discrimination in this cohort and should be confirmed in larger studies before being used to estimate risk for individual patients.

### 4.5. Comparison with Previous Studies

Our results should be placed in the context of the wider literature with some care. Recent meta-analyses have summarized the diagnostic performance of ultrasound-based AI models for thyroid nodules, including studies with external validation datasets and direct comparisons with radiologists [10,11,12]. These reviews report generally good diagnostic performance but also considerable heterogeneity between studies. A meta-analysis focusing specifically on cytologically indeterminate nodules reported a pooled AUC of 0.82 (95% CI 0.81–0.84) across machine learning and deep learning models, with very high heterogeneity among deep learning models (I^2^ = 99.7%) [13], and an external validation study reported AUCs of 0.69–0.70 for deep learning models, similar to those of experienced radiologists [17]. Other studies have compared AI systems with radiologists and have shown that AI assistance can help less experienced radiologists reach an accuracy similar to that of senior readers [18,19,20].

These pooled estimates come mostly from broader or mixed populations and should not be compared numerically with the present small surgical cohort of Bethesda III–IV nodules. They do, however, highlight two points that also apply to our study. First, performance measured in a single center may not be maintained on data from other institutions and ultrasound platforms. Second, although no patient of the present cohort contributed to model development, hospitals of the same group did, and a single site does not by itself establish generalizability, which will require validation in multiple independent centers.

### 4.6. Clinical Implications and Communication of Uncertainty

TRAICK is intended to provide additional information for preoperative decision-making, not to replace FNAB or clinical judgment. In practice, AI-derived risk categories will sometimes agree and sometimes conflict with cytology and with the radiologist’s assessment. Patients with indeterminate cytology already face considerable uncertainty, and discordant information may increase rather than reduce this uncertainty if it is not communicated carefully. Structured approaches to communicating uncertainty in indeterminate thyroid cytology have recently been proposed [21], and AI-derived information should be incorporated into such shared decision-making rather than presented as an additional diagnosis.

The present study did not assess whether TRAICK changes clinical decisions, repeat biopsy rates, surgery rates, or patient outcomes. These potential benefits remain hypotheses. They should be tested in prospective studies of management impact, and decision-curve or net-benefit analyses in larger, unselected cohorts would help to define the range of risk thresholds at which the system might be clinically useful. In addition, the predictive values observed here reflect the malignancy rate of this surgical cohort (44.2%), which is higher than the expected risk of malignancy of Bethesda III–IV nodules [7], and would be expected to differ in routine practice.

### 4.7. Strengths and Limitations

The strengths of this study include evaluation of the model in patients who were not part of its development data, the use of postoperative histopathology of the index nodule as the reference standard for all nodules, separate evaluation of the two TRAICK outputs with prespecified thresholds, blinded reading by two radiologists, and sensitivity analyses addressing the main sources of possible misclassification of the reference standard.

This study also has several limitations. First, it was a small, single-center, retrospective study. The confidence intervals were wide, the paired comparisons were exploratory and not adjusted for multiple testing, and several subgroups contained very few nodules; the subgroup results, in particular, may be overinterpreted and should be read with caution. Second, only operated nodules were included. This selected surgical cohort had a malignancy rate of 44.2%, higher than expected for Bethesda III–IV nodules, and spectrum bias may have affected sensitivity and specificity as well as the predictive values; 35 of the 93 nodules with indeterminate cytology were not operated on and could not be evaluated. Third, only two readers took part, one of whom was involved in the development of TRAICK, and the readings were based on a single selected static image per nodule, which may have limited the assessment of features such as shape that are usually judged on more than one plane. Fourth, the reference standard has its own uncertainties. The classification of low-risk neoplasms such as NIFTP differs between studies, carcinoma was found outside the index nodule in six cases, and interobserver variability among pathologists was not assessed; our sensitivity analyses addressed the first two of these issues but cannot remove them completely. Fifth, all examinations were performed on a single ultrasound platform, and the study cannot establish how TRAICK would perform with other devices, institutions, or populations. In addition, the validation set of the development data was not separated at the patient level, and the internal test set came from hospitals that also contributed training images, so the internal test performance reported in File S2 may be optimistic; this does not apply to the present cohort, whose patients were not part of the development data. However, because hospitals of the same group, with similar equipment and imaging protocols, contributed development data, the present results may overestimate performance at unrelated institutions. Sixth, TRAICK reports only final categories, so ROC analyses were based on ordinal categories rather than a continuous score, and the optimal threshold for the Bethesda-like output was not defined. Seventh, no formal assessment of algorithmic bias or fairness, for example across age groups or sexes, was possible in this small sample. Finally, clinical impact was not evaluated.

## 5. Conclusions

In this small, selected surgical cohort of cytologically indeterminate thyroid nodules, the AUC of the ACR TI-RADS output of TRAICK did not differ significantly from that of the more accurate of two radiologists, and its specificity was higher; compared with the second radiologist, its accuracy, specificity, and AUC were higher. Its Bethesda-like output showed promising discrimination across categories, although sensitivity at the prespecified threshold was moderate. These findings suggest that TRAICK has the potential to provide supplementary information to support preoperative decision-making in indeterminate thyroid nodules. They do not show superiority over radiologists or FNAB, generalizability to other settings, or a reduction in repeat biopsies or surgery. Independent prospective multicenter studies, including an assessment of clinical impact, are needed before clinical use.

## Figures and Tables

**Table 1:** Diagnostic performance of the radiologists and the two TRAICK outputs, with index-nodule histopathology as the reference standard (*n* = 52; 23 malignant, 29 nonmalignant).

Reader/Output	Accuracy	Sensitivity	Specificity	PPV	NPV	κ vs. Histology (95% CI)	AUC (95% CI)
Radiologist 1 (≥TR4)	32/52 61.5% (48.0–73.5)	21/23 91.3% (73.2–97.6)	11/29 37.9% (22.7–56.0)	21/39 53.8% (38.6–68.4)	11/13 84.6% (57.8–95.7)	0.273 (0.067 to 0.479)	0.777 (0.658–0.895)
Radiologist 2 (≥TR4)	27/52 51.9% (38.7–64.9)	20/23 87.0% (67.9–95.5)	7/29 24.1% (12.2–42.1)	20/42 47.6% (33.4–62.3)	7/10 70.0% (39.7–89.2)	0.102 (−0.091 to 0.296)	0.629 (0.489–0.769)
TRAICK ACR TI-RADS (≥TR4)	37/52 71.2% (57.7–81.7)	18/23 78.3% (58.1–90.3)	19/29 65.5% (47.3–80.1)	18/28 64.3% (45.8–79.3)	19/24 79.2% (59.5–90.8)	0.428 (0.188 to 0.668)	0.762 (0.640–0.884)
TRAICK Bethesda-like (≥V)	39/52 75.0% (61.8–84.8)	14/23 60.9% (40.8–77.8)	25/29 86.2% (69.4–94.5)	14/18 77.8% (54.8–91.0)	25/34 73.5% (56.9–85.4)	0.482 (0.244 to 0.719)	0.822 (0.707–0.938)

Values are *n*/*N* and % (95% CI). Malignant: carcinoma in the index nodule; nonmalignant: benign lesions and WHO 2022 low-risk neoplasms (NIFTP, *n* = 6; WT-UMP, *n* = 1). CIs for proportions: Wilson score method; κ: Fleiss–Cohen–Everitt asymptotic standard error; AUC: DeLong method, calculated from the ordinal categories (ACR TI-RADS TR2–TR5; Bethesda-like II–VI). Test-positive: ACR TI-RADS TR4–TR5 for the radiologists and the TRAICK ACR TI-RADS output; categories V–VI for the TRAICK Bethesda-like output. Paired comparisons with the TRAICK ACR TI-RADS output: vs. Radiologist 1, accuracy *p* = 0.267, specificity *p* = 0.021, AUC *p* = 0.783; vs. Radiologist 2, accuracy *p* = 0.013, specificity *p* < 0.001, AUC *p* = 0.042 (exact McNemar test; DeLong test). TRAICK Bethesda-like AUC vs. Radiologist 1 *p* = 0.573, vs. Radiologist 2 *p* = 0.029, vs. TRAICK ACR TI-RADS *p* = 0.444. Full results are given in Supplementary Table S1. κ, Cohen’s kappa; NIFTP, noninvasive follicular thyroid neoplasm with papillary-like nuclear features; WT-UMP, well-differentiated tumor of uncertain malignant potential; PPV, positive predictive value; NPV, negative predictive value; AUC, area under the ROC curve.

**Table 2:** Agreement between readers in ACR TI-RADS categorization (*n* = 52).

Comparison	Exact Agreement	Unweighted κ (95% CI)	Interpretation *	Linear-Weighted κ (95% CI)	Binary κ, ≥TR4 (95% CI)
Radiologist 1 vs. Radiologist 2	33/52 63.5% (49.9–75.2)	0.411 (0.194 to 0.628)	Moderate	0.506 (0.315 to 0.697)	0.611 (0.353 to 0.869)
Radiologist 1 vs. TRAICK ACR TI-RADS	31/52 59.6% (46.1–71.8)	0.391 (0.196 to 0.586)	Fair	0.496 (0.327 to 0.666)	0.480 (0.261 to 0.699)
Radiologist 2 vs. TRAICK ACR TI-RADS	29/52 55.8% (42.3–68.4)	0.336 (0.150 to 0.523)	Fair	0.379 (0.201 to 0.557)	0.435 (0.226 to 0.644)

κ values with 95% CI (Fleiss–Cohen–Everitt asymptotic standard error). * Landis and Koch: 0.21–0.40 fair, 0.41–0.60 moderate. Unweighted κ is calculated across ACR TI-RADS categories and is the main estimate; *p* < 0.001 for all unweighted κ values (test of κ = 0).

**Table 3:** Reclassification of cytologically indeterminate nodules by the TRAICK Bethesda-like output, by index-nodule histopathology.

Cytological Bethesda (FNAB)	Bethesda-like II	Bethesda-like III	Bethesda-like IV	Bethesda-like V	Bethesda-like VI	Total
Bethesda III	9 1 M/8 N	4 0 M/4 N	4 1 M/3 N	5 3 M/2 N	7 7 M/0 N	29 12 M/17 N
Bethesda IV	4 1 M/3 N	3 0 M/3 N	10 6 M/4 N	4 3 M/1 N	2 1 M/1 N	23 11 M/12 N
Malignancy rate in TRAICK Bethesda-like category, % (95% CI)	2/13 15.4% (4.3–42.2)	0/7 0.0% (0.0–35.4)	7/14 50.0% (26.8–73.2)	6/9 66.7% (35.4–87.9)	8/9 88.9% (56.5–98.0)	23/52 44.2%

Each cell gives the number of nodules, split into malignant (M) and nonmalignant (N) on index-nodule histopathology. Columns show the TRAICK Bethesda-like category. The last row gives the malignancy rate in each TRAICK Bethesda-like category, with Wilson 95% CIs. This table describes reclassification within an indeterminate cytology cohort and is not a comparison of diagnostic accuracy between TRAICK and FNAB. FNAB, fine-needle aspiration biopsy.

**Table 4:** Malignancy rate by TRAICK ACR TI-RADS category within each cytological Bethesda category.

Cytological Bethesda	TRAICK ACR TI-RADS	Malignant/*n*	Malignancy Rate, % (95% CI)	*p* for Trend †
Bethesda III	TR3	1/12	8.3% (1.5–35.4)	<0.001
Bethesda III	TR4	6/12	50.0% (25.4–74.6)	
Bethesda III	TR5	5/5	100.0% (56.6–100.0)	
Bethesda IV	TR2	0/1	0.0% (0.0–79.3)	0.132
Bethesda IV	TR3	4/11	36.4% (15.2–64.6)	
Bethesda IV	TR4	5/8	62.5% (30.6–86.3)	
Bethesda IV	TR5	2/3	66.7% (20.8–93.9)	

*n*, number of nodules in each subgroup. Rates are given with Wilson 95% CIs. † Cochran–Armitage test for trend across TRAICK ACR TI-RADS categories within each cytology group. Subgroups with fewer than 10 nodules give wide CIs, and their rates should be read with caution.

## Data Availability

The data presented in this study are available on reasonable request from the corresponding author. The data are not publicly available because they contain patient information. TRAICK outputs for the 52 nodules are available from the corresponding author on reasonable request; the TRAICK software (version 1.9.4; TRAICK, Istanbul, Türkiye) is not publicly available. The R code used for the statistical analysis is available from the corresponding author on reasonable request.

## Supplementary Materials

The following supporting information can be downloaded at: https://www.mdpi.com/article/10.3390/diagnostics16213616/s1, Table S1: Paired comparisons between readers on the same 52 nodules; Table S2: Sensitivity analyses; File S1: STARD-AI checklist; File S2: TRAICK AI System—model development summary.
