# Supplementary material for: Artificial Intelligence–Assisted ACR TI-RADS and Bethesda-like Risk Prediction for Indeterminate Thyroid Nodules: A Retrospective Proof-of-Concept Evaluation
Source: Diagnostics (Basel). 2026 Nov 2;16(21):3616. doi: 10.3390/diagnostics16213616 (PMC13762284; doi:10.3390/diagnostics16213616)
Supplement: Supplementary file 1 [file diagnostics-16-03616-s001.zip › Supplementary File S2.pdf]

Supplementary Materials. TRAICK AI System – Model Development and Validation Summary

1. Overview and Purpose

The TRAICK AI System (version 1.9.4) was developed as a multi-stage deep-learning platform designed for automated segmentation and classification of thyroid nodules on gray-scale ultrasound images. This Supplementary Materials describes the data processing pipeline, model architecture, training strategy, and feature-level performance metrics that underlie the retrospective proof-of-concept evaluation reported in the main manuscript.

2. Training Dataset and Preprocessing

A total of 9,975 gray-scale ultrasound images (720 × 640 pixels) were retrospectively collected from several institutions (including Istanbul Medeniyet University Göztepe Training and Research Hospital and hospitals of the healthcare group to which the institution of the evaluation cohort belongs). Images showing the fine-needle aspiration needle inside the nodule, and images flagged as ineligible during annotation, were excluded, yielding a final dataset of 9,185 high-quality images for model training and evaluation.

Prior to model input, all images underwent standardized preprocessing including gray-scale normalization, resizing (to 224 × 224 or 256 × 256 pixels for the feature classifiers and 640 × 640 pixels for the segmentation model), and automated removal of non-diagnostic elements. The development data were partitioned into training, validation, and test subsets (approximately 52%, 22%, and 26% of the annotated nodule images). The test subset was separated at the patient level, so that no test patient contributed images to the training or validation subsets; the validation subset was drawn at random from the remaining images, so some patients contributed images to both the training and validation subsets. This split applies to the ACR TI-RADS feature classifiers; the segmentation model was developed with a separate, image-level split (Section 7). Hospitals of the same healthcare group, including the hospital of the proof-of-concept evaluation cohort reported in the main manuscript, contributed development images from other patients. No patient from that cohort contributed images to the development dataset, and the cohort was not used for training, validation, or testing.

The development images were acquired between 2013 and 2023, mostly with Toshiba Aplio 500 and GE LOGIQ S8 scanners; about 92% of the images with a recorded transducer type were obtained with a linear probe.

3. Annotation and Labeling

All images were manually annotated by expert radiologists to delineate nodule boundaries and identify the five core ACR TI-RADS features: composition, echogenicity, shape, margin, and echogenic foci. Annotations were stored in formats compatible with the deep-learning frameworks used during model development.

To address class imbalance, the feature classifiers were trained with classical augmentation only (random rotation of up to ±15°, horizontal and vertical flips and, for some models, brightness and contrast changes), and minority classes were oversampled. Generative augmentation was used for the segmentation model only: about 1,019 synthetic images produced with a Pix2Pix generative adversarial network were added to its training set. Augmented and synthetic images were used for training only; no augmented or synthetic image was included in the validation or test sets.

4. Model Architecture

The TRAICK AI System comprises two integrated modules. The first module performs automated nodule segmentation using a YOLOv8-based architecture, generating a nodule-specific region of interest for downstream analysis. The second module consists of five dedicated feature-classification submodels, each trained independently to predict one of the five ACR TI-RADS features.

The segmentation model (YOLOv8-L instance segmentation, input 640 × 640 pixels) was trained from scratch. Each feature classifier receives a two-channel input: the full anonymized frame and the same frame with the pixels outside the nodule mask set to zero. During training, the mask was taken from the annotator's outline; in use, it is taken from the output of the segmentation model.

Feature-level outputs were converted into ACR TI-RADS point values according to the official ACR scoring definitions and summed to produce a total ACR TI-RADS score, which was then mapped to a final ACR TI-RADS category (TR1–5) using standard ACR thresholds (TR1 = 0 points, TR2 = 2 points, TR3 = 3 points, TR4 = 4–6 points, TR5 ≥ 7 points). This hybrid design preserved model interpretability while ensuring strict adherence to ACR TI-RADS guidelines.

Each feature-specific classifier was initialized with ImageNet-pretrained weights to improve convergence and generalization; for the hybrid vision-transformer/ResNet models, only the convolutional (ResNet) part was pretrained. To address class imbalance across feature categories, focal loss was employed during optimization.

The models were implemented in PyTorch (versions 2.0–2.5) with PyTorch Lightning and the timm library; the segmentation model was trained with Ultralytics 8.2.4. The feature classifiers were optimized with RAdam or AdamW (initial learning rates between 1.5×10^{-4} and 1.9×10^{-3}, with cosine or reduce-on-plateau schedules) at a batch size of 64, for up to 150 epochs, with early stopping when the validation micro-F1 had not improved for 10–30 epochs; the epoch with the best validation micro-F1 was kept. Echogenic foci were treated as a multi-label task with a sigmoid focal loss. Contrast-limited adaptive histogram equalization (CLAHE) was applied for some classifiers. The segmentation model was trained for 50 epochs with AdamW (learning rate 3×10^{-4} decaying to 3×10^{-6}) at a batch size of 8, and the epoch with the best validation score was kept. Training was performed on single NVIDIA GPUs (Tesla P100, Tesla T4, RTX 3060 and RTX 3070).

Table S1. TRAICK AI System feature-to-score mapping based on ACR TI-RADS definitions.

ACR TI-RADS Feature	Predicted Class (AI output)	ACR TI-RADS Equivalent Category	Assigned Points
**Composition (4-class)**	Cystic / Almost cystic	Benign	0
	Spongiform	Benign	0
	Mixed solid–cystic	Intermediary	1
	Solid	Suspicious	2
**Echogenicity (4-class)**	Anechoic	Benign	0
	Hyperechoic / Isoechoic	Low-risk	1
	Mildly hypoechoic	Intermediate	2
	Markedly hypoechoic	High-risk	3
**Shape (binary)**	Wider-than-tall	Benign	0
	Taller-than-wide	Suspicious	3
**Margin (4-class)**	Smooth	Benign	0
	Ill-defined	Low-risk	0
	Lobulated / Irregular	Suspicious	2
	Extra-thyroidal extension	High-risk	3
**Echogenic foci (multi-label)**	None / Large comet-tail artifact	Benign	0
	Macrocalcification	Suspicious	1
	Peripheral (rim) calcification	Suspicious	2

	Punctate echogenic foci (microcalcification)	High-risk	3

5. Bethesda Classification Module

The TRAICK AI System was trained to perform multi-class Bethesda classification (categories II–VI) using a hybrid labeling strategy that incorporated both cytological and histopathological reference standards. During model training, nodules without surgical confirmation (Bethesda II–III) were labeled according to their FNAB results, whereas nodules with Bethesda V–VI cytology that were confirmed malignant at surgery were labeled using final histopathology. For Bethesda IV nodules, labels were harmonized based on postoperative diagnoses to reduce ambiguity in this indeterminate category.

Model optimization employed weighted categorical cross-entropy to penalize misclassification of clinically high-risk categories. Class weights were assigned inversely to class prevalence, with Bethesda V and VI receiving the highest weights due to their low frequency and high clinical importance.

The Bethesda-like category is predicted directly from the image by this separate classifier; it is not derived from the ACR TI-RADS feature outputs. The model gives a probability for each category, and the category with the highest probability is reported; no thresholds are applied.

This labeling strategy reflects real-world diagnostic pathways but introduces an inherent limitation: benign nodule labels rely on FNAB cytology, which may yield false-negative results in lesions with malignant potential (e.g., minimally invasive follicular carcinoma or microcarcinoma). Consequently, the training dataset contains asymmetric label certainty, with histopathological labels providing higher certainty for malignant classes.

6. Feature-Level Classification Performance

Independent classification models were trained for each of the five ACR TI-RADS features. Performance metrics for each feature-specific model on the held-out test set are summarized in Table S2.

Table S2. Summary of TRAICK v1.9.4 performance by ACR TI-RADS feature on the held-out test set.

ACR TI-RADS Feature	Best Model	Test F1	Sensitivity	Specificity
Echogenicity	ConvNeXt-Base	0.747	0.747	0.916
Composition	ConvNeXt-Base	0.888	0.888	0.963
Margin	CSPResNeXt50	0.591	0.591	0.863
Echogenic Foci	ConvNeXt-Base	0.838	0.806	0.954
Shape	ViT-small/ResNet50d	0.693	0.655	0.874

F1 and sensitivity are micro-averaged across classes; for features with mutually exclusive classes they are therefore equal to the overall accuracy. Specificity is micro-averaged in a one-vs-rest manner.

Composition and echogenic foci classification demonstrated the highest performance, with test F1 scores of 0.888 and 0.838, respectively. Margin classification showed comparatively lower performance (F1 = 0.591), consistent with the known subjectivity of margin assessment in thyroid ultrasound. Shape classification, treated as a binary task, achieved a test F1 of 0.693.

7. Segmentation Performance

No test-set metric was recorded during training of the segmentation model. On its validation set (796 images, 846 nodules), mask mAP50 was 0.913 and mAP50–95 was 0.636; because the same images were used to select the best training epoch, these values are probably optimistic. In a later internal test on 227 images from the segmentation test split, compared with the annotators' outlines, the mean Dice similarity coefficient was 0.826 (95% CI 0.789–0.859; median 0.918), the mean intersection over union was 0.757 (95% CI 0.720–0.790), and the nodule was detected in 214 of 227 images (94.3%). Because the segmentation data were split at the image level, some patients also contributed other images to the training set; in the 78 images from patients without any training or validation image, the mean Dice coefficient was 0.819 (95% CI 0.759–0.871).
