# Supplementary material for: Artificial Intelligence–Assisted ACR TI-RADS and Bethesda-like Risk Prediction for Indeterminate Thyroid Nodules: A Retrospective Proof-of-Concept Evaluation
Source: Diagnostics (Basel). 2026 Nov 2;16(21):3616. doi: 10.3390/diagnostics16213616 (PMC13762284; doi:10.3390/diagnostics16213616)
Supplement: Supplementary file 1 [file diagnostics-16-03616-s001.zip › Supplementary_File_S1_STARD-AI_checklist.pdf]

Supplementary File S1. STARD-AI checklist

Artificial Intelligence–Assisted ACR TI-RADS and Bethesda-like Risk Prediction for Indeterminate Thyroid Nodules: A Retrospective Proof-of-Concept Evaluation

Checklist items follow the STARD-AI reporting guideline (Sounderajah V, et al. Nat Med. 2025;31:3283–3289). Item descriptions are summarized; see the original publication for the full wording. Page and line numbers refer to the accepted manuscript file with continuous line numbering; section names follow the published article.

Section / topic	No.	Checklist item (summary)	Reported in (section)	Page / line
**Title or abstract**	1	Identification as a diagnostic accuracy study of an AI index test, reporting at least one measure of accuracy	Title; Abstract	p. 1, l. 3; p. 4, ll. 65–90
**Abstract**	2	Structured summary of study design, methods, results, and conclusions	Abstract	p. 4, ll. 65–90
**Introduction**	3	Scientific and clinical background, intended use and clinical role of the index test, novelty, and place in the clinical workflow	Introduction, paragraphs 1–3	p. 5, ll. 98–125
	4	Study objectives and hypotheses	Introduction, last paragraph	p. 5, ll. 126–130
**Methods – Study design**	5	Whether data collection was prospective or retrospective	Methods, Study design and reporting	p. 6, ll. 134–136
**Methods – Ethics**	6	Ethics committee approval, or justification if not required	Methods, Study design and reporting; Institutional Review Board Statement	p. 6, ll. 139–144; pp. 19–20, ll. 614–618
**Methods – Participants**	7	Eligibility criteria (inclusion and exclusion), in the order applied, at participant and data level	Methods, Patient selection; Image selection	p. 6, ll. 146–160; p. 7, ll. 185–192
	8	How potentially eligible participants were identified	Methods, Patient selection	p. 6, ll. 146–148
	9	Where and when potentially eligible participants were identified (setting, location, dates)	Methods, Patient selection	p. 6, ll. 146–148
	10	Whether participants formed a consecutive, random, or convenience series	Methods, Patient selection	p. 6, ll. 146–148
**Methods – Dataset**	11	Source of the data (routinely collected, collected for the study, or open repository)	Methods, Image selection	p. 7, ll. 185–192
	12	Who annotated the dataset, their expertise, and how annotation was performed	File S2, Section 3 (development data); not applicable to the test set (reference standard = histopathology)	File S2, Section 3
	13	Capture devices (manufacturer, model) and software (with version) used for the index test	Methods, Ultrasound examination and FNAB; TRAICK AI System	p. 7, ll. 173–174; p. 8, l. 238
	14	Data acquisition protocol and pre-processing, in enough detail for replication	Methods, Ultrasound examination and FNAB; Image selection; TRAICK AI System; File S2	p. 7, ll. 173–176; p. 7, ll. 185–192; p. 9, ll. 267–269; File S2,

Section / topic	No.	Checklist item (summary)	Reported in (section)	Page / line
				Sections 2 and 4
**Methods – Test methods**	15a	Index test described in enough detail to allow replication	Methods, TRAICK AI System; Figures 2–4; File S2	pp. 8–9, ll. 238–271; Figures 2–4; File S2, Sections 4 and 5
	15b	How the index test was developed (training, validation, testing, external evaluation), with sample sizes	Methods, TRAICK AI System; File S2	p. 9, ll. 246–265; File S2, Sections 2–7
	15c	Definition and rationale for test positivity cut-offs or categories, prespecified vs exploratory	Methods, Definitions and analysis plan	p. 9, ll. 273–280
	15d	Intended end user of the index test and the level of expertise required	Methods, TRAICK AI System	p. 8, ll. 243–245
	16a	Reference standard described in enough detail to allow replication	Methods, Histopathological reference standard	pp. 7–8, ll. 206–215
	16b	Rationale for choosing the reference standard	Methods, Histopathological reference standard	p. 7, l. 206
	16c	Definition and rationale for reference standard categories, prespecified vs exploratory	Methods, Histopathological reference standard; Imaging–pathology correlation in multifocal disease	pp. 7–8, ll. 206–221; p. 8, ll. 223–236
	17a	Whether clinical information and reference standard results were available to index test performers/readers	Methods, Image selection; Radiologist reading; TRAICK AI System	p. 7, ll. 190–191; p. 7, ll. 197–198
	17b	Whether clinical information and index test results were available to reference standard assessors	Methods, Ultrasound examination and FNAB; Histopathological reference standard	p. 7, ll. 182–183; pp. 7–8, ll. 207–208
**Methods – Analysis**	18	Methods for estimating or comparing measures of diagnostic accuracy	Methods, Statistical analysis	pp. 10–11, ll. 287–325
	19	How indeterminate index test or reference standard results were handled	Methods, Patient selection; Statistical analysis	p. 6, ll. 154–158; p. 10, ll. 298–300
	20	How missing data on the index test and reference standard were handled	Methods, Statistical analysis	p. 10, ll. 298–300
	21	Analyses of variability in diagnostic accuracy, prespecified vs exploratory	Methods, Statistical analysis (sensitivity analyses); Results, Sensitivity analyses; Supplementary Table S2	p. 10, ll. 313–318; pp. 13–14, ll. 418–432; Supplementary Table S2
	22	Intended sample size and how it was determined	Methods, Statistical analysis	p. 10, ll. 319–321

Section / topic	No.	Checklist item (summary)	Reported in (section)	Page / line
	23	Performance error analysis and algorithmic bias/fairness assessment, if done	Results, Paired comparisons; Supplementary Table S1; Discussion, Strengths and limitations	p. 12, ll. 365–371; p. 18, ll. 582–583; Supplementary Table S1
**Results – Participants and dataset**	24	Flow of participants, using a diagram	Results, Study population; Figure 1	p. 11, ll. 329–330; Figure 1
	25	Baseline demographic, clinical, and technical characteristics of the datasets	Results, Study population; File S2	p. 11, ll. 329–333; File S2, Section 2
	26a	Distribution of severity of disease in those with the target condition	Results, Study population (histological subtypes)	p. 11, ll. 337–340
	26b	Distribution of alternative diagnoses in those without the target condition	Results, Study population	p. 11, ll. 340–343
	27	Time interval and any clinical interventions between index test and reference standard	Results, Study population	p. 11, ll. 333–334
	28	Whether the dataset reflects the distribution of the target condition in the intended-use population	Methods, Patient selection (paragraph 2); Discussion, Limitations	p. 6, ll. 162–168; p. 18, ll. 561–565
	29	For external evaluation, how the evaluation dataset differs from the development datasets	Methods, TRAICK AI System	p. 9, ll. 253–258
**Results – Test results**	30	Cross-tabulation of index test results (or their distribution) by reference standard results	Results; Figure 5; Table 3; Figure 7; Table 4	p. 11, l. 349; p. 13, ll. 396–416; Figures 5 and 7; Tables 3 and 4
	31	Estimates of diagnostic accuracy and their precision (e.g., 95% CI)	Results; Table 1; Figure 6; Table 2; Supplementary Tables S1–S2	pp. 11–13, ll. 352–394; Figure 6; Tables 1–2; Supplementary Tables S1–S2
	32	Any adverse events from performing the index test or the reference standard	Methods, Study design and reporting	p. 6, ll. 141–142
**Discussion**	33	Limitations, including sources of bias, statistical uncertainty, and generalizability	Discussion, Strengths and limitations	pp. 17–18, ll. 553–584
	34	Implications for practice, including intended use and clinical role of the index test	Discussion; Conclusions	p. 17, ll. 537–551; p. 19, ll. 586–595
	35	Ethical considerations and fairness issues related to the index test	Discussion, Strengths and limitations	p. 18, ll. 582–583
**Other information**	36	Registration number and name of registry	Institutional Review Board Statement	p. 20, ll. 616–618

Section / topic	No.	Checklist item (summary)	Reported in (section)	Page / line
	37	Where the full study protocol can be accessed	Institutional Review Board Statement	p. 20, ll. 616–618
	38	Sources of funding and other support; role of funders	Funding	p. 19, ll. 612–613
	39	Commercial interests, if applicable	Conflicts of Interest	p. 19, ll. 603–611
	40a	Availability of datasets and code, and any restrictions on reuse	Data Availability Statement	p. 21, ll. 619–623
	40b	Whether index test outputs are stored, auditable, and available for evaluation	Data Availability Statement	p. 21, ll. 621–622
